# Hétérogénéité de la transmission du paludisme urbain dans les districts sanitaires de Bouaké, Côte d’Ivoire

**DOI:** 10.48327/mtsi.v6i2.2026.731

**Published:** 2026-04-26

**Authors:** Konan Fabrice ASSOUHO, Négnorogo GUINDO-COULIBALY, Dounin Danielle ZOH, Konan Rodolphe Mardoché AZONGNIBO, Akré Maurice ADJA

**Affiliations:** 1 UFR Agriculture, Ressources halieutiques et agro-industries, Université de San Pedro, San Pedro, Côte d’Ivoire; 2 Institut Pierre Richet (IPR) / Institut national de santé publique (INSP), Bouaké, Côte d’Ivoire; 3 UFR Biosciences, Université Félix Houphouët-Boigny, Abidjan, Côte d’Ivoire; 4 Département de sciences et techniques, Université Alassane Ouattara, Bouaké, Côte d’Ivoire; 5 Institut de géographie tropicale, Université Félix Houphouët-Boigny, Abidjan, Côte d’Ivoire

**Keywords:** *Anopheles gambiae*, *Plasmodium falciparum*, Paludisme, Transmission, Milieu urbain, Bouaké, Côte d’Ivoire, Afrique subsaharienne, *Anopheles gambiae*, *Plasmodium falciparum*, Malaria, Transmission, Urban environment, Bouaké, Côte d'Ivoire, Sub-Saharan Africa

## Abstract

**Justification:**

Des travaux réalisés dans la ville de Bouaké il y a environ 20 ans ont montré un fort niveau de transmission du paludisme essentiellement dû au vecteur *Anopheles gambiae*, avec une recrudescence manifestée en saison pluvieuse. Des efforts de lutte à travers la distribution de moustiquaires imprégnées d’insecticides à longue durée d’action (MILDA) ont été menés par le Programme national de lutte contre le paludisme. Cependant, la crise politico-militaire de 2002 à 2011 a provoqué un important flux migratoire des habitants de cette ville, laissant le milieu naturel préservé. Le retour des populations à Bouaké a donc nécessité la mise en place de nouveaux programmes de lutte avec l’utilisation des MILDA. De nouvelles études de recherche sont maintenant nécessaires afin de recueillir des données sur les paramètres de transmission du paludisme. La présente étude s’inscrit dans ce contexte. Elle a pour objectif d’évaluer la transmission vectorielle du paludisme à travers la détermination de paramètres entomologiques dans les trois districts sanitaires de la ville de Bouaké.

**Matériel et méthodes:**

Les investigations ont été menées en milieu urbain pendant la saison pluvieuse, du 10 au 29 juin 2016, dans les trois districts sanitaires de Bouaké (Nord-Ouest, Nord-Est, Sud). Les moustiques ont été collectés par captures sur sujet humain et identifiés. Le comportement trophique des femelles vectrices a été examiné et les paramètres entomologiques de la transmission du paludisme ont été estimés. L’âge physiologique des femelles vectrices a été déterminé après la dissection de leurs ovaires et les moustiques infestés par *Plasmodium falciparum* ont été détectés par le test ELISA-CSP.

**Résultats:**

*An. gambiae* a été la seule espèce vectrice du paludisme collectée. Elle a présenté une tendance à l’endophagie et un taux moyen de parturité supérieur à 90 % dans tous les districts. Les densités agressives de l’espèce étaient respectivement de 3,6, 15,5 et 14,4 piqûres/homme/nuit (p/h/n) dans les districts Nord-Ouest, Nord-Est et Sud. Les taux d’infestation étaient en moyenne de 4,2 %, 2,9 % et 1,9 % pour les districts Nord-Ouest, Nord-Est et Sud avec des taux d’inoculation entomologique de 0,15, 0,45 et 0,22 p/h/n respectivement.

**Conclusion:**

La transmission du paludisme existe dans les trois districts de la ville de Bouaké avec un niveau deux fois supérieur dans le district Nord-Est. Cette transmission est assurée par l’espèce *An. gambiae* qui a présenté une tendance à l’endophagie dans tous les sites.

## Abstract (en)

**Rationale:**

Approximately 20 years ago, studies conducted in the city of Bouaké revealed a high level of malaria transmission, primarily due to the *Anopheles gambiae* vector increasing during the rainy season. The National Malaria Control Program implemented control efforts involving the distribution of long-lasting insecticidal nets (LLINs). However, the political and military crisis from 2002 to 2011 caused many residents to leave the city, leaving the natural environment largely undisturbed. The subsequent return of the population to Bouaké necessitated implementing new LLIN-based control programs. New research studies are needed to collect data on malaria transmission parameters. This study is conducted within this context. The objective is to assess vector-borne malaria transmission by determining entomological parameters in the city's three health districts.

**Materials and methods:**

Investigations were conducted in urban areas during the rainy season from June 10 to 29, 2016, in Bouaké's three health districts (Northwest, Northeast, and South). Mosquitoes were collected from human-biting samples and identified. We examined the feeding behavior of female vectors and estimated the entomological parameters of malaria transmission. We determined the physiological age of the female vectors by dissecting their ovaries and detected mosquitoes infected with *Plasmodium falciparum* using the ELISA-CSP test.

**Results:**

Only the malaria vector species *An. gambiae* was collected. The species exhibited a tendency toward endophagy and had an average parturition rate exceeding 90% in all districts. Aggressive densities of the species were 3.6, 15.5, and 14.4 bites/ person/night (b/p/n) in the Northwest, Northeast, and South districts, respectively. The infection rates were 4.2%, 2.9%, and 1.9% in the Northwest, Northeast, and South districts, respectively, with entomological inoculation rates of 0.15, 0.45, and 0.22 b/p/n.

**Conclusion:**

Malaria transmission occurs in all three districts of Bouaké, with levels twice as high in the Northeast District. This transmission is carried out by the *An. gambiae* species, which exhibited a tendency toward endophagous feeding at all sites.

**Figure:**
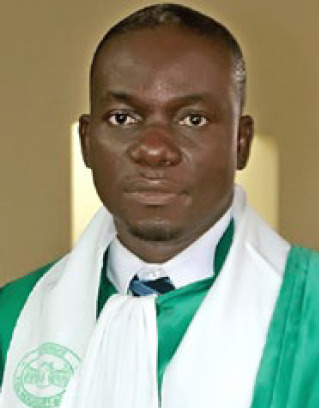

## Introduction

Depuis 2000, on estime que 2,3 milliards de cas de paludisme et 14 millions de décès ont été évités dans le monde entier dont 1 million de vies sauvées sur la seule année 2024. Les progrès pour atteindre les objectifs mondiaux d’élimination de la maladie se sont poursuivis, 47 pays et un territoire étant désormais officiellement certifiés exempts de paludisme par l’OMS [28]. Malgré ces progrès, le paludisme reste un problème de santé mondial important, avec environ 282 millions de cas et 610 000 décès en 2024, soit environ 9 millions de cas de plus que l’année précédente. La région Afrique de l’OMS continue de supporter la plus grande partie du fardeau, avec 11 pays représentant environ les deux tiers des cas et des décès dans le monde [28]. Les progrès en matière de réduction du taux de mortalité lié au paludisme restent toutefois très insuffisants. Ainsi, malgré des efforts importants et divers plans de contrôle, le paludisme reste un défi majeur pour la santé publique, en particulier en Afrique sub-saharienne [17,27].

En Côte d’Ivoire, comme dans la plupart des grands pays d’Afrique, le paludisme est la cause majeure de morbidité et de mortalité et constitue le premier motif de consultation et d’hospitalisation [21,7]. Se trouvant dans une zone d’endémie palustre, ce pays connaît une transmission permanente du paludisme tout au long de l’année avec une recrudescence pendant la saison pluvieuse [3,14,26]. À Bouaké, ville située au centre de la Côte d’Ivoire, la transmission est liée aux conditions climatiques, écologiques, hydrographiques et aux activités humaines [2,10,14]. À ces facteurs, s’ajoutent des situations particulières telles que l’introduction de la riziculture irriguée et intensive et l’apparition de la résistance des vecteurs du paludisme aux insecticides. D’après les travaux de Dossou-Yovo *et al.* [14] réalisés sur toute l’année dans cette ville il y a environ 20 ans, *An. gambiae* s.l*.* était le principal vecteur du paludisme (représentant plus de 98 % de la faune anophélienne) avec un taux considérable d’inoculation entomologique (TIE) de 339 piqûres infestantes/homme/an et des pics en saison des pluies. Ce taux essentiellement dû à *An. gambiae* était influencé par une importante saison des pluies conduisant à une recrudescence en début de saison pluvieuse [20]. Par ailleurs, des travaux réalisés après la crise militaro-politique de 2002 à 2011 en Côte d’Ivoire par Adja *et al.* [1] et Zoh *et al.* [36], visant à actualiser les données de la transmission vectorielle dans cette ville, ont également montré que cette espèce était toujours le principal vecteur du paludisme à Bouaké avec une forte agressivité en saison pluvieuse.

Des efforts de lutte et de prévention contre le paludisme ont été menés à travers la distribution en masse de moustiquaires imprégnées d’insecticides à longue durée d’action (MILDA) utilisées comme moyen de protection. Ces efforts menés par le ministère de la Santé et de l’hygiène publique à travers le Programme national de lutte contre le paludisme (PNLP) visaient une couverture de toute la population. La crise de 2002 à 2011 a provoqué un important flux migratoire des habitants de cette ville. Plusieurs quartiers ont été désertés laissant le milieu naturel sans entretien et l’arrêt de toute activité anti-vectorielle. Le retour des populations dans ces quartiers a nécessité la mise en place de programmes de recherche destinés à recueillir de nouvelles données sur les paramètres de transmission et la répartition du paludisme. L’intérêt de cette étude réalisée en zone urbaine pendant la saison des pluies est qu’elle cible la période la plus propice au développement des vecteurs du paludisme à Bouaké, c’est à dire la période de transmission intense de la maladie. Elle a donc pour objectif d’évaluer le niveau de la transmission vectorielle du paludisme par la détermination des paramètres entomologiques dans les quartiers des trois districts sanitaires de Bouaké.

## Matériel et méthodes

### Milieu d’étude

Bouaké (7°40’47’’N, 5°1’38’’O) est une ville située au centre de la Côte d’Ivoire à 372 km d’Abidjan. Avec une superficie de 312 km^2^ et une population de plus de 500 000 habitants, Bouaké représente la deuxième ville de la Côte d’Ivoire après Abidjan, la capitale économique. Elle est située dans une zone de transition écologique (forêt/savane). Les pluies s’étalent généralement sur 8 mois, de mars à octobre [16]. Les moyennes pluviométriques annuelles oscillent entre 1 000 et 1 200 mm. La température varie peu au cours de l’année, avec des moyennes de 28 à 32 °C. L’humidité relative annuelle est comprise entre 75 et 90 %. Par sa forme vallonnée, la ville abrite plusieurs kilomètres de rubans marécageux. L’espace urbain est sillonné par de nombreux petits cours d’eau espacés de 500 à 800 m les uns des autres. Cette situation fait que tous les quartiers de la ville, à l’exception du centre commercial, sont traversés ou limités par des rubans de bas-fonds humides.

La ville de Bouaké est divisée en trois districts sanitaires : le district Nord-Ouest, le district Nord-Est et le district Sud. La zone d’étude, la conception de l’étude et l’épidémiologie locale du paludisme ont déjà été décrites en détail [33]. Pour chaque district, trois quartiers ont été choisis. Pour le district Nord-Ouest, les quartiers concernés sont N’Gattakro, Djézoukouamékro et Dar-Es-Salam; Sokoura, Belle Ville et Attienkro pour le district Nord-Est; et pour le district Sud, Kennedy, Air France et N’Gouatanoukro **(**Fig. 11). Dans cette région, la transmission du paludisme est intense, *Plasmodium falciparum* étant la principale espèce parasitaire [32] et *An. gambiae* s.l. le principal vecteur [24].

**Figure 1:**
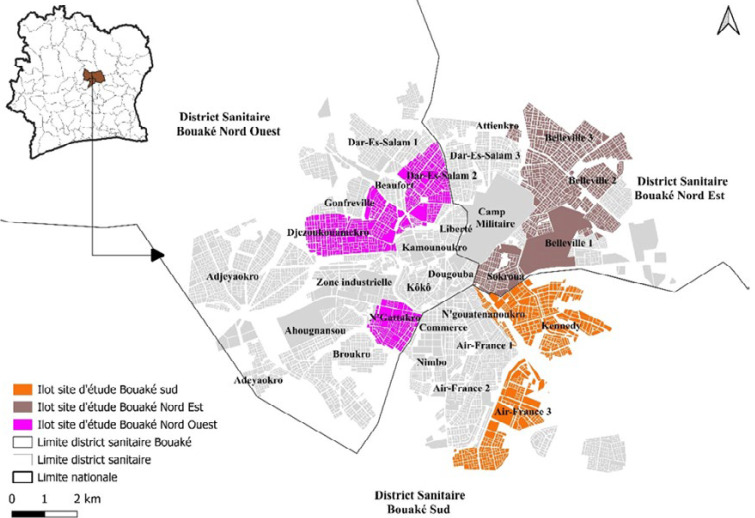
Zone de Bouaké présentant les sites d’étude (source : CNTIG, 2018 / Palevalute, 2018 – Réalisation : AZONGNIBO Mardoché, 2024)

### Échantillonnage des moustiques sur le terrain

L’échantillonnage a été effectué en milieu urbain pendant la saison pluvieuse, du 10 au 29 juin 2016, dans les trois districts sanitaires de Bouaké. Le choix des points de capture a commencé par une visite préliminaire dans ces districts. À partir du plan de la ville et en tenant compte de la présence de l’environnement des districts, trois quartiers par district ont été choisis, puis chaque quartier a été divisé en différents lots appelés points de capture. La collecte des moustiques a été entreprise à l’aide de la méthode de capture sur sujet humain (CSH). Pendant trois nuits consécutives (de 17 h à 9 h), des CSH ont été réalisées dans quatre points de capture par quartier, avec deux collecteurs dont un à l’intérieur et l’autre à l’extérieur des habitations sélectionnées (soit 216 séances de capture).

Ces captures ont été réalisées par deux équipes de bénévoles de tout sexe (résidents locaux). La première équipe a travaillé de 17 h à minuit et la seconde de minuit à 9 h. Les moustiques capturés ont été regroupés toutes les heures par site et conservés dans des sacs séparés. Grâce à cette stratégie, la densité agressive ou densité d’anophèles antropophiles par personne et par unité de temps (nuit) a été estimée et utilisée pour le calcul des TIE. Tous les moustiques collectés ont été identifiés morphologiquement à l’aide de la clé d’identification des Culicidae d’Edwards [15] et de celle des anophèles femelles afrotropicales [9]. Les ovaires des femelles d’anophèles ont été disséqués et le degré d’enroulement des trachéoles ovariennes a été observé pour déterminer leur statut de parité sur la base des critères de Detinova [11]. Toutes les femelles anophèles collectées ont été conservées individuellement dans des tubes Eppendorf, contenant un déshydratant, étiquetés avec le nom du site d’étude, le point et la date de collecte. Elles ont été conservées à -20 °C pour des analyses ultérieures au laboratoire de l’Institut Pierre Richet de Bouaké.

### Traitement des moustiques au laboratoire

Un test *Enzyme-linked immunosorbent assay* (ELISA) a été utilisé pour tester la présence de protéine circumsporozoïte (CSP) de *P. falciparum* (ce parasite représente l’espèce prédominante et la cause de plus de 95 % des cas de paludisme). La tête et le thorax des femelles anophèles ont été utilisés pour le test. Ces deux parties ont été séparées du reste du corps et homogénéisées dans un tampon de blocage (caséine à 0,5 %, NaOH 0,1 N, PBS 1×). Les procédures utilisées étaient celles de Burkot *et al.* [6] et Wirtz *et al*. [35]. Un échantillon de moustique était positif si la valeur de la densité optique (DO) était supérieure à deux fois la DO moyenne de quatre puits de contrôle négatif (moustiques non infectés) sur la plaque ELISA.

### Paramètres entomologiques et analyse statistique

La saisie des données quantitatives recueillies au cours des enquêtes entomologiques a été effectuée sous Excel et la base a été transférée dans la version 8.0 du logiciel STATA pour analyse. La densité agressive, le taux d’infestation et de parturité ont été calculés en utilisant les méthodes standards déjà décrites par Fontenille *et al.* [19]. Pour la comparaison des densités agressives moyennes, il a été utilisé le test de Kruskal-Wallis. Le test de chi^2^ a permis de comparer les taux d’infestation et de parturité. Toutes les analyses ont été réalisées avec un intervalle de confiance de 5 %.

## Résultats

### Faune culicidienne

Au total, 29 528 moustiques ont été capturés dans les trois districts par 216 hommes/nuit, soit une nuisance culicidienne de 136,7 piqûres/homme/ nuit (p/h/n). Ces moustiques sont répartis en quatre genres (*Anopheles*, *Culex*, *Mansonia* et *Aedes*), tous retrouvés dans chacun des districts*.* Les captures ont permis de récolter dans le district Nord-Ouest, 6 104 moustiques répartis en 8 espèces (*An. gambiae, An. pharoensis, Cx. quinquefasciatus, Cx. nebulosus, Cx. annulioris, Cx. cinereus, Man. africana* et *Ae. aegypti)* avec une nuisance culicidienne de 84,7 p/h/n. Dans les trois quartiers du district, les nuisances culcidiennes ont été respectivement de 70,7, 85,9 et 97,6 p/h/n respectivement à Dar-Es-Salam, Djezoukouamekro et N’Gattakro. Le genre *Culex* a été prédominant avec une proportion de 95,2 % de la faune suivi du genre *Anopheles* avec 4,3 % (Tableau I). *Cx. quinquefasciatus* a été l’espèce prédominante avec une proportion de 97,7 %.

**Tableau I:** Faune culicidienne des sites échantillonnés des districts sanitaires de Bouaké

**Genre**	**Espèce**	**Bouaké Nord-Ouest**			**Bouaké Nord-Est**			**Bouaké Sud**		
		Dar-Es-Salam n (%)	Djézoukouamékro n (%)	N'Gattakro n (%)	Sokoura n (%)	Attienkro n (%)	Belle Ville n (%)	Air France n (%)	Kennedy n (%)	N'Goutanoukro n (%)
Anopheles	*An. gambiae*	35 (2,1)	185 (9)	41 (1,7)	76 (1,7)	585 (15,8)	456 (24,8)	21 (0,4)	987 (32,4)	28 (0,5)
	*An. pharoensis*	-	3 (0,1)	-	1 (0,02)	6 (0,2)	-	-	8 (0,3)	-
	*An. ziemanni*	-	-	-	-	-	-	-	1 (0,0)	-
**Total 1**		**35 (2,1)**	**186 (9,1)**	**41 (1,7)**	**77 (1,7)**	**588 (16)**	**464 (24,8)**	**21 (0,4)**	**996 (32,7)**	**28 (0,5)**
Culex	*Cx. quinquefasciatus*	1 644 (96,9)	1 868 (90,5)	2 271 (96,9)	4 464 (98,2)	1 592 (43,0)	1 320 (71,7)	4 714 (99,4)	1 564 (51,3)	5 512 (99,5)
	*Cx. nebulosus*	6 (0,4)	1 (0,05)	-	-	-	7 (0,4)	-	18 (0,6)	-
	*Cx. annulioris*	-	1 (0,05)	2 (0,1)	-	1 (0,03)	-	-	1 (0,03)	-
	*Cx. decens*	-	-	-	-	8 (0,2)	5 (0,3)	-	-	-
	*Cx. cinereus*	8 (0,5)	3 (0,1)	6 (0,3)	3 (0,1)	-	7 (0,4)	-	-	1 (0,02)
	*Cx. ingrami*	-	-	-	-	-	25 (1,4)	-	-	-
	*Cx. tigripes*	-	-	-	-	-	6 (0,3)	-	-	-
**Total 2**		**1 658 (97,7)**	**1 873 (90,8)**	**2 276 (97,2)**	**4 467 (98,3)**	**3 701 (43,2)**	**1 370 (74,4)**	**4 714 (99,4)**	**1 583 (51,9)**	**5 513 (99,5)**
Mansonia	*Man. africana*	-	2 (0,1)	-	1 (0,02)	333 (9,1)	1 (0,1)	-	94 (3,1)	-
	*Man. uniformis*	-	-	-	-	840 (22,7)	12 (0,7)	-	55 (1,8)	-
**Total 3**		-	**2 (0,1)**	-	**1 (0,0)**	**1 173 (31,7)**	**13 (0,7)**	-	**149 (4,9)**	-
Aedes	*Ae. aegypti*	4 (0,2)	-	24 (1,0)	-	338 (9,1)	2 (0,1)	7 (0,1)	319 (10,5)	1 (0,02)
	*Ae. vittatus*	-	-	-	-	-	-	-	2 (0,1)	-
	*Ae. palpalis*	-	-	-	-	-	-	-	1 (0,03)	-
	*Ae. africanus*	-	-	-	-	1 (0,03)	-	-	-	-
**Total 4**		**0,2**	-	**24 (1,0)**	-	**339 (9,2)**	**2 (0,1)**	**7 (0,1)**	**322 (10,6)**	**1 (0,02)**
**TOTAL**		**1 697**	**2 063**	**2 344**	**4 545**	**3 704**	**1 841**	**4 742**	**3 003**	**5 542**

Au total, 10 090 moustiques ont été récoltés dans le district de Bouaké Nord-Est. Le genre *Culex* a été prédominant (73,7 %), suivi des genres *Mansonia* (11,8 %) et *Anopheles* (11,1 %). Les captures sur homme ont permis de répertorier 13 espèces avec une dominance de *Cx. quinquefasciatus* (73,1 % des captures). La nuisance culicidienne moyenne a été de 140,1 p/h/n dont 189,4 p/h/n à Sokoura, 154,3 p/h/n à Attienkro et 76,7 p/h/n à Belle Ville. Dans les 3 quartiers, le genre *Culex* a été prédominant, avec 98,3 %, 74,4 % et 43,2 % respectivement à Sokoura, Belle Ville et Attienkro. Il a été suivi du genre *Mansonia* (31,7 % à Attienkro) et du genre *Anopheles* avec des proportions de 24,8 % et 1,7 % respectivement à Belle Ville et Sokoura (Tableau I).

Un total de 13 334 moustiques a été capturé dans le district de Bouaké Sud. Comme dans les 2 autres districts, le genre *Culex* a été majoritaire (88,6 % des moustiques). *Cx. quinquefasciatus* a été prédominant parmi les 12 espèces collectées, avec une proportion de 88,4 %. La nuisance moyenne du district a été de 185,2 p/h/n avec des nuisances de 197,6, 230,9 et 127,1 p/h/n respectivement à Air France, N’Gouatanoukro et Kennedy (Tableau I). Le genre *Culex* a été majoritairement représenté dans chacun des 3 quartiers du district à des proportions de 99,5 %, 99,4 % et 51,9 % respectivement pour N’Gouatanoukro, Air France et Kennedy. Dans ce district, le genre *Anopheles* a représenté entre 0,4 et 32,7 % des culicidés.

### Comportement de piqûre d’*An. gambiae* s.l.

L’organisation concomitante des captures à l’intérieur et à l’extérieur des habitations a permis de déterminer les habitudes trophiques des femelles d’*An. gambiae* s.l*.* (Tableau II). Le taux d’endopha-gie ou d’exophagie est la proportion de femelles de moustiques vecteurs du paludisme qui piquent à l’intérieur ou à l’extérieur des habitations. Il s’exprime en pourcentage. Dans la ville de Bouaké, sur les 2 414 femelles d’*An. gambiae* s.l. capturées, 1 269 (52,6 %) l’ont été à l’intérieur et 1 145 (47,4 %) à l’extérieur des habitations. Cependant, au niveau du district sanitaire de Bouaké Nord-Ouest, les femelles d’*An. gambiae* se nourrissent aussi bien à l’intérieur qu’à l’extérieur des habitations. Il a été observé à Dar-Es-Salam et à Djézoukouamékro une exophagie. Dans les quartiers de Sokoura et Air France, bien que l’on observe en général une endophagie, les femelles d’*An. gambiae* se nourrissent aussi bien à l’intérieur qu’à l’extérieur des habitations.

**Tableau II:** Comportement de piqûres des femelles d’*An. gambiae* s.l. dans les trois districts sanitaires de Bouaké

Districts	Quartiers	Femelles capturées		Statut majoritaire
		Int n (%)	Ext n (%)	
**Bouaké Nord-Ouest**	N'Guattakro	31 (75,60)	10 (24,4)	Endophagie
	Dar-Es-Salam	12 (34,3)	23 (65,7)	Exophagie
	Djézoukouamékro	84 (45,9)	99 (54,1)	Exophagie
**TOTAL**		**127 (49)**	**132 (51)**	**Endophagie**
**Bouaké Nord-Est**	Sokoura	38 (50)	38 (50)	Endo-Exophagie
	Belle Ville	261 (56,2)	203 (43,8)	Endophagie
	Attienkro	306 (52,6)	276 (47,4)	Endophagie
**TOTAL**		**605 (53,9)**	**517 (46,1)**	**Endophagie**
**Bouaké Sud**	Air France	10 (50)	10 (50)	Endo-Exophagie
	Kennedy	497 (52,9)	443 (47,1)	Endophagie
	N'Goutanoukro	11 (42,3)	15 (57,7)	Exophagie
**TOTAL**		**518 (52,5)**	**468 (47,5)**	**Endophagie**

n : Effectif; Int : Intérieur; Ext : Extérieur; (%) : Pourcentage de femelles dans le total capturé

### Densité agressive d’*An. gambiae* s.l.

*An. gambiae* s.l. a été le seul vecteur du paludisme identifié dans les trois districts de la ville de Bouaké. Sa densité agressive moyenne a été de 11,2 p/h/n sur l’ensemble de la ville (Tableau III). Dans le district Nord-Ouest, cette densité a été de 3,6 p/h/n. En considérant les quartiers de ce district, la densité agressive obtenue à Djézoukouamekro (7,7 p/h/n) a été significativement supérieure à celles de N’Gattakro (1,7 p/h/n) et Dar-Es-Salam (1,5 p/h/n) (p=0,002).

**Tableau III:** Densité agressive d’*An. gambiae* s.l. dans les trois districts de Bouaké

Districts	Quartiers	ma (p/h/n)	IC
**Bouaké Nord-Ouest**	N'Gattakro	1,7 (41)	0,8 - 2,6
	Dar-Es-Salam	1,5 (35)	0,5 - 2,4
	Djézoukouamékro	7,7 (185)	4,1 - 11,3
**Moyenne**	**3,6 (261)**	**2,2 - 5**	
**Bouaké Nord-Est**	Belle Ville	19 (456)	13,7 - 24,3
	Attienkro	24,4 (585)	16,6 - 32,2
	Sokoura	32 (76)	1,8 - 4,5
**Moyenne**	**15,5 (1 117)**	**11,8 - 19,2**	
**Bouaké Sud**	Air France	0,9 (21)	0,5 - 1,3
	Kennedy	41,1 (987)	28,5 - 53,8
	N'Goutanoukro	1,2 (28)	0,5 - 1,8
**Moyenne**	**14,4 (1 036)**	**8,4 - 20,4**	

ma : agressivité en piqûres par humain et par nuit (p/h/n); IC : intervalle de confiance (95 %) /

La densité agressive d’*An. gambiae* s.l. à Bouaké Nord-Est a été de 15,5 p/h/n. La densité agressive du vecteur observée à Sokoura (3,2 p/h/n) a été largement inférieure à celles d’Attienkro (24,4 p/h/n) et de Belle Ville (19 p/h/n) (p < 0,10^4^). Dans le district Sud de Bouaké, l’agressivité d’*An. gambiae* a été de 14,4 p/h/n. La densité agressive observée à Kennedy (41,1 p/h/n) a été largement plus élevée (p < 0,0001) que celle estimée à Air France (0,9 p/h/n) et N’Gouatanoukro (1,2 p/h/n). L’agressivité d’*An*. *gambiae* s.l. à Bouaké Nord-Ouest a été significativement inférieure (p < 0,0001) à celle observée dans les deux autres districts où les densités n’ont pas significativement varié.

### *Taux de parturité d’*An. gambiae *s.l.*

Dans l’ensemble, 809 femelles d’*An. gambiae* (216 dans le district Nord-Ouest, 377 dans le district Nord-Est et 216 dans le district Sud) ont été disséquées sur place avec un taux de parturité estimé à 93,8 %. Ce taux était estimé respectivement à 92,6 % et 95 % à l’intérieur et à l’extérieur des habitations (Tableau IV).

**Tableau IV:** Taux de parturité des femelles d’An. gambiae récoltées dans les trois districts de Bouaké

Districts	Quartiers	Nombre de testées		Nombre de pares		Taux de parturitié		TP total
		Int n (%)	Ext n (%)	Int n (%)	Ext n (%)	Int n (%)	Ext n (%)	
**Bouaké Nord-Ouest**	Dar-Es-Salam	11	19	11	19	100	100	100
	Dézoukouamékro	83	80	77	73	92,8	91,3	92
	N'Gattakro	15	8	15	8	100	100	100
**TOTAL**		109	107	103	100	94,5	93,5	94
**Bouaké Nord-Est**	Sokoura	30	40	28	34	93,3	85	88,6
	Attienkro	68	86	68	86	100	100	100
	Belle Ville	97	56	83	55	85,6	98,2	90,2
**TOTAL**		195	182	179	175	91,8	96,2	94,9
**Bouaké Sud**	N'Goutanoukro	13	14	8	11	61,5	78,6	70,4
	Air France	9	13	7	9	77,8	69,2	72,7
	Kennedy	65	102	65	102	100	100	100
**TOTAL**		87	129	80	122	92	94,6	93,5

TP : Taux de parturité; Int : Intérieur; Ext : Extérieur

Dans le district sanitaire de Bouaké Nord-Ouest, 216 femelles d’*An. gambiae* ont été disséquées avec un taux de parturité estimé à 94 %. Ce taux était presque identique à l’intérieur et à l’extérieur des habitations avec des proportions respectives de 94,5 % et 93,5 %. Dans les quartiers de Dar-Es-Salam et N’Gattakro, le taux de parturité des a été de 100 % et de 92 % à Djézoukouamékro.

Dans l’ensemble, 377 femelles d’*An. gambiae* ont été disséquées avec un taux de parturité estimé à 94,9 % dans le district de Bouaké Nord-Est. À l’intérieur et l’extérieur des habitations de ce district, les taux de parturité étaient respectivement de 91,8 % et 96,2 %. À Attienkro, le taux de parturité des femelles d’*An. gambiae* était de 100 %, celui de Sokoura et de Belle Ville respectivement de 88,6 % et 90,2 %.

Dans le district de Bouaké Sud, 216 femelles d’*An. gambiae* ont été disséquées avec un taux de parturité estimé à 93,5 %. Les taux de parturité à l’intérieur et à l’extérieur des habitations ont été respectivement de 92 % et 94,6 %. *À Kennedy,* ce taux a été de 100 %. N’Gouatanoukro et Air France ont eu des taux de parturité presque identiques estimés respectivement à 70,4 % et 72,7 %.

### Taux d’infection et taux d’inoculation entomologique

Sur 813 femelles d’anophèles testées pour l’ensemble de la ville de Bouaké, 24 étaient infectées. Le taux moyen d’infestation était de 3 % avec un TIE de 0,33 p/h/n (Tableau V). Le taux d’infestation du district Nord-Ouest était de 4,2 % sans différence significative entre ce taux et celui des districts Nord-Est (2,9 %; p = 0,40) et Sud (1,8%; p = 0,14).

**Tableau V:** Taux d’infection et taux d’inoculation entomologique

Districts	Quartiers	Nb testé (Nb infecté)			TIE (pi/h/n)
		Int n (%)	Ext n (%)	Total	
**Bouaké Nord-Ouest**	N'Gattakro	14 (1)	8 (1)	22 (2)	0,15
	Djezoukouamékro	85 (5)	78 (1)	163 (6)	0,28
	Dar-Es-Salam	11 (0)	19 (1)	30 (1)	0,05
**TOTAL**		**110 (6)**	**105 (3)**	**215 (9)**	**0,15**
**Bouaké Nord-Est**	Attienkro	85 (3)	63 (1)	148 (4)	0,67
	Sokoura	38 (1)	35 (0)	73 (1)	0,04
	Belle Ville	106 (4)	52 (2)	158 (6)	0,72
**TOTAL**		**229 (8)**	**150 (3)**	**379 (11)**	**0,45**
**Bouaké Sud**	Kennedy	70 (1)	99 (2)	169 (3)	0,45
	Air France	12 (0)	11 (1)	23 (1)	0,004
	N'Gouattanoukro	10 (0)	17 (0)	27 (0)	0
**TOTAL**		**92 (1)**	**127 (3)**	**219 (4)**	**0,22**
**TOTAL BOUAKÉ**		**431 (15)**	**382 (9)**	**813 (24)**	**0,33**

Nb testé : nombre de femelles testées; Nb infecté: nombre de femelles infectées; TIE : taux d’inoculation entomologique; pi/h/n : piqûres infestées par homme et par nuit

Dans le district Nord-Ouest, 9 femelles d’anophèles ont été trouvées infectées par *P. falciparum* sur les 215 moustiques femelles testées, soit un taux d’infestation de 4,2 %. Les taux d’infestation des femelles d’anophèles ont été de 3,7 % à Djézoukouamékro, 3,33 % à Dar-Es-Salam et de 9,1 % à N’Gattakro. Ces taux ne présentent pas de différence significative (p = 0,41). Les analyses réalisées sur les anophèles capturées à l’intérieur et à l’extérieur des habitations n’ont pas montré de différences significatives entre le taux d’infestation dans les quartiers de Djézoukouamékro (p = 0,11), de N’Gattakro (p = 0,67) et de Dar-Es-Salam (p = 0,43). Le TIE dans ce district est de 0,15 p/h/n. Cependant, le quartier de Djézoukouamékro a présenté le taux le plus élevé (0,28 p/h/n). Les taux des deux autres quartiers (N’Gattakro et Dar-Es-Salam) étaient respectivement de 0,15 p/h/n et 0,05 p/h/n.

Sur un ensemble de 379 femelles testées, 11 moustiques étaient infectés dans le district Nord-Est, soit un taux d’infestation de 2,9 %. Les taux d’infestation de Belle Ville, d’Attienkro et de Sokoura ont été respectivement de 3,8 %, 2,7 % et 1,3 %. Ils n’ont pas présenté de différence significative (p = 0,59). Les taux d’infestation obtenus à l’intérieur et à l’extérieur des maisons n’ont montré aucune différence sensible à Belle Ville (p = 0,98), à Sokoura (p = 0,33) et à Attienkro p = 0,47). Le TIE a été faible à Sokoura (0,04 p/h/n) comparativement aux deux autres quartiers où des TIE de 0,67 p/h/n et 0,72 p/h/n ont été obtenus respectivement à Attienkro et Belle Ville.

L’analyse des 219 femelles d’anophèles récoltées dans le district Sud a confirmé l’infection de 4 moustiques soit un taux d’infestation de 1,8 %. Le taux d’infestation moyen des femelles capturées à Kennedy a été de 1,8 %, comparable à celui estimé à Air France : 4,3 % (p = 0,41). Les femelles capturées à N’Gouatanoukro n’ont présenté aucune infection. Dans ces 2 quartiers (Kennedy et Air France), les taux d’infestation des femelles récoltées à l’intérieur et à l’extérieur des habitations sont similaires (p = 0,28). Le quartier de Kennedy a enregistré un fort TIE de 0,45 p/h/n.

## Discussion

L’inventaire de la faune culicidienne anthropophile montre une grande diversité et une forte abondance de moustiques en saison pluvieuse. Quatre genres et 16 espèces de moustiques ont été collectés par la méthode de capture sur sujet humain dans l’ensemble des trois districts de Bouaké. Cette diversification de faune culicidienne dans tous les districts peut s’expliquer par la diversité de biotopes rencontrée dans cette localité qui offre des gîtes spécifiques favorables au développement de chaque espèce : rizières, flaques d’eau, mares, marécages, collections d’eau dans les vieux récipients, etc. [1]. L’importance des populations culicidiennes est intimement liée au fonctionnement et à la dynamique des gîtes larvaires [5]. Les gîtes (mares, pneus, récipients) changent au fil du temps (saisons, pluies) et de l’espace (urbain/rural) en termes de nombre, de type, de productivité en larves, et d’influence des facteurs physiques (température, oxygène, pH) pour la reproduction des moustiques [25]. Les résultats entomologiques de CSH montraient que la faune culicidienne anthropophile dans la ville de Bouaké était essentiellement dominée par l’espèce *Cx. quinquefasciatus* comme observé par Adja *et al.* [1]. La prédominance de cette espèce est souvent associée au milieu urbain [3]. Dans le cas de Bouaké, elle constitue une grande source de nuisance pour les populations. Les travaux réalisés par Klinkenberg *et al.* [23] à Accra et ceux de Fillinger *et al.* [18] à Dar-Es-Salam sont en adéquation avec ces observations.

Dans les trois districts sanitaires, la faune anophélienne était majoritairement dominée par l’espèce *An. gambiae* s.l*.,* principal vecteur du paludisme. Cette espèce occupe aussi la deuxième place au sein de la faune culicidienne par sa fréquence dans les captures au niveau de tous les districts après *Cx. quinquefasciatus*. Cette situation est conforme aux travaux de Assouho *et al*., [3], qui confirment sa présence dans les villes. La représentativité marquante d’*An. gambiae* est aussi associée aux actions humaines favorisant la présence de gîtes permanents ou sub-permanents, tels que ceux créés par l’irrigation des cultures, les rizières ou l’urbanisation [7,25,27,31]. Par ailleurs, la présence d’anophèles est associée également à la détérioration de l’environnement causée par la guerre. Leur présence est souvent liée à des conditions environnementales dégradées, que les guerres peuvent aggraver : création de gîtes larvaires (eau stagnante due aux destructions, absence de gestion de l’eau); destruction des infrastructures sanitaires; perturbation des programmes de lutte, favorisant ainsi la prolifération des moustiques vecteurs de paludisme. Ce cycle vicieux accentue les problèmes sanitaires dans les zones de conflit [2].

Dans l’ensemble, les investigations révèlent que les moustiques ont tendance à se gorger davantage à l’intérieur des habitations qu’à l’extérieur dans les districts Nord-Est et Sud. C’est une endophagie malgré l’introduction de MILDA dans les habitations. Elle pourrait s’expliquer par la forte résistance aux insecticides pyréthrinoïdes utilisés pour traiter les MILDA, mais soulève également des questions sur l’utilisation réelle de ces moustiquaires par les populations pour éviter les piqûres des moustiques vecteurs et prévenir le paludisme. Par ailleurs, ce comportement du vecteur semble bien adapté aux habitudes nocturnes des humains, imposées par la saison des pluies. Une telle adaptation du comportement de piqûre du moustique aux habitudes nocturnes des humains en milieu urbain est similaire aux observations faites par Salako *et al.* [30] au Bénin. Par contre, dans le district Nord-Ouest, les populations vectrices du paludisme ont une tendance à se nourrir aussi bien à l’intérieur qu’à l’extérieur, ce qui reflète le début d’un changement dans le comportement des vecteurs.

La densité agressive d’*An. gambiae* s.l. a été globalement plus élevée dans les districts Nord-Est et Sud que dans le district Nord-Ouest. Cette différence pourrait résulter de l’aménagement des bas-fonds pour la riziculture irriguée (Attienkro et Belle Ville), le maraîchage (Kennedy) et la détérioration de l’environnement causée par la guerre. À Kennedy, la densité agressive moyenne de 41,2 p/h/n pour *An. gambiae* a été largement supérieure à celle trouvée par Dossou-Yovo *et al*. [14] qui avaient obtenu pour cette même période une densité agressive de 18,5 p/h/n pour *An. gambiae*. Par ailleurs, d’autres études ont également révélé une densité plus forte de moustiques dans les zones de cultures irriguées (rizières, jardins maraîchers) que dans les zones voisines sans cultures ou sans cultures irriguées [12,14,22]. Les quartiers Djézoukouamékro, N’Gattakro et Dar-Es-Salam du district Nord-Ouest, de par leur position géographique, représentent en grande partie le centre de la ville de Bouaké, ce qui explique la faible densité agressive dans ce district. Ces observations confirment les travaux antérieurs qui ont montré une réduction de la densité d’*An. gambiae* de la périphérie vers le centre-ville [4,13]. Les populations de moustiques du complexe *An. gambiae* s.l., vecteurs du paludisme, sont plus abondantes dans les zones périphériques (rurales ou périurbaines). Elles diminuent progressivement à mesure que l’on s’approche du centre-ville [2], probablement en raison de différences environnementales (moins de sites de reproduction, plus d’urbanisation, meilleure gestion de l’eau, etc.) ou de stratégies de contrôle du paludisme.

Les taux de parturité observés pour cette période d’étude sont très élevés dans les trois districts de Bouaké. Cela indique un fort vieillissement de la faune anophélienne, donc une forte proportion de femelles potentiellement dangereuses car le plus souvent infectieuses, et qui ont plus de temps pour être en contact avec des humains avant de mourir. Le taux de femelles pares est de 100 % dans les quartiers de Kennedy, Dar-Es-Salam et Attienkro. Ainsi, pour ces quartiers répartis dans les trois districts de la ville, la fraction de la population d’*An. gambiae* qui peut transmettre le paludisme est identique. Par ailleurs, Dossou-Yovo *et al*. [14] signalaient un taux de parturité moyen en saison des pluies de 71,7 % pour *An. gambiae* à Kennedy, alors que notre étude montre un taux supérieur (100 %) pour la même espèce et la même saison pluvieuse environ 25 ans plus tard.

Le taux d’infestation, très élevé dans l’ensemble des districts, est probablement dû aux taux de parturité élevés. En effet, plus les moustiques sont âgés, plus leur susceptibilité d’héberger le parasite est grande. Il pourrait en résulter une transmission permanente et intense du paludisme. L’endémicité du paludisme avec une recrudescence pendant la saison des pluies dans la ville de Bouaké est semblable à celle habituellement décrite en Côte d’Ivoire [3].

Le TIE de 0,33 p/h/n (soit 9,9 p/h/mois) que nous avons observé était inférieur à celui de Dossou-Yovo *et al*. [14], qui était de 1,7 p/h/n soit 51 p/h/ mois. La situation est très hétérogène à Bouaké comme dans la plupart des villes d’Afrique [8,14,34]. Chaque quartier constitue un cas particulier avec ses influences propres qui peuvent dépendre des humains ou des caractéristiques naturelles du milieu. La diversité de situations qui est observée illustre bien l’importance des conditions locales, parfois même micro-écologiques, sur l’intensité de la transmission du paludisme. Le risque d’être infecté par les *Plasmodium* reste important à Bouaké.

## Conclusion

Le présent travail réalisé dans la ville de Bouaké a révélé une transmission du paludisme hétérogène, causée par *An. gambiae*, dont le comportement endophage en fait le seul vecteur du paludisme dans les différents quartiers de la ville. Ce vecteur est prédominant dans le district Nord-Est, dans les quartiers d’Attienkro et de Belle Ville. Le quartier de Kennedy présente la population *An. gambiae* la plus élevée et demeure la source d’enracinement du vecteur pour le district Sud. Dans l’ensemble des districts échantillonnés dans la ville de Bouaké, le risque de transmission entomologique le plus élevé a été observé dans le district Nord-Est, notamment dans les quartiers d’Attienkro et de Belle Ville. Ce district présente un niveau de risque de transmission du paludisme deux fois supérieur à celui du district du Sud et trois fois supérieur à celui du district Ouest. Il mérite donc une attention particulière dans le cadre des programmes de lutte contre le paludisme dans cette ville. Par ailleurs, des actions de lutte telles que l’utilisation des larvicides sont à encourager chez les personnes pratiquant des activités comme le maraîchage ou la riziculture.

## Conflit d’intérêt, financement et principes éthiques

Cette recherche a bénéficié d’un financement de l’Institut de recherche pour le développement (IRD) à travers le projet JEAI EVAPALCI. Les auteurs ne déclarent aucun conflit d’intérêt. L’étude a reçu les approbations du Comité national d’éthique et de recherche de Côte d’Ivoire (Juin 2014; No. 41/MSLS/CNER-dkn) ainsi que des autorités sanitaires de la ville de Bouaké. L’autorisation d’enquête a été obtenue auprès des chefs de quartiers et des propriétaires de maison ayant servi de point de capture. De plus, le consentement de la communauté a été obtenu au préalable dans tous les sites. Les collecteurs volontaires de moustiques ont donné leur consentement oral avant de participer à l’étude. Ils ont également été soumis à des visites médicales régulières avec un traitement préventif contre le paludisme conformément aux recommandations du PNLP de Côte d’Ivoire. Ils étaient tous vaccinés contre la fièvre jaune.

## Remerciements

Nous exprimons notre gratitude à la Direction régionale de la santé de Bouaké pour l’opportunité qu’elle nous a donnée en nous autorisant à enquêter dans les différents quartiers de la ville. Nous sommes très reconnaissants aux chefs des quartiers échantillonnés ainsi qu’à l’ensemble des collecteurs de moustiques. Enfin, nous remercions également M. Jean-Luc Amany pour son appui lors de la réalisation de ce travail, et l’IRD à travers le projet de la JEAI EVAPALCI pour le soutien financier.

## Contribution des auteurs et autrices

KFA, AMA, NGC ont participé à l’élaboration du sujet et l’écriture du manuscrit. KFA, DDZ et NGC ont participé à l’enquête. KFA et KRMA ont conduit la saisie et l’analyse des données. KFA et DDZ ont participé à la rédaction du manuscrit. KFA, AMA et NGC ont participé à la correction du manuscrit et tous les auteurs ont donné leur accord pour la publication du manuscrit.

## Authors’ contributions

KFA, AMA, and NGC contributed to the development of the topic and the writing of the manuscript. KFA, DDZ, and NGC participated in the survey. KFA and KRMA conducted data entry and analysis. KFA and DDZ contributed to the drafting of the manuscript. KFA, AMA, and NGC participated in the revision of the manuscript, and all authors approved the manuscript for publication.
