# Supplementary material for: Variability in urban malaria transmission across health districts in Bouaké, Côte d’Ivoire
Source: Med Trop Sante Int. 2026 Apr 26;6(2):mtsi.v6i2.2026.731. [Article in French] doi: 10.48327/mtsi.v6i2.2026.731 (PMC13423299; doi:10.48327/mtsi.v6i2.2026.731)
Supplement: Related texts

# Research article: Variability in urban malaria transmission across health districts in Bouaké, Côte d’Ivoire

## Introduction

Since 2000, an estimated 2.3 billion malaria cases and 14 million malaria-related deaths have been prevented worldwide. In 2024 alone, 1 million lives were saved. Progress toward achieving global malaria elimination targets has continued, with 47 countries and one territory now officially certified malaria-free by the WHO [28]. However, malaria remains a significant global health issue, with approximately 282 million cases and 610,000 deaths in 2024—approximately 9 million more cases than the previous year. The WHO African Region continues to bear the greatest burden, with 11 countries accounting for approximately two-thirds of global cases and deaths [28]. However, progress in reducing malaria-related mortality remains insufficient. Thus, despite significant efforts and various control programs, malaria remains a major public health challenge, particularly in sub-Saharan Africa [17,27].

In Côte d’Ivoire, as in most large African countries, malaria is the leading cause of morbidity and mortality, as well as the primary reason for medical consultations and hospitalizations [21,7]. The country is located in a malaria-endemic zone and experiences year-round malaria transmission, with an increase during the rainy season [3,14,26]. In Bouaké, a city in central Côte d’Ivoire, malaria transmission is linked to climatic, ecological, hydrographic, and human activity conditions [2,10,14]. In addition to these factors, there are specific circumstances, such as the introduction of irrigated, intensive rice farming, and the emergence of insecticide resistance among malaria vectors. According to Dossou-Yovo *et al.*’s work [14] conducted throughout the year in Bouaké about 20 years ago, *An. gambiae* s.l. was the primary malaria vector, accounting for over 98% of the *Anopheles* population, with a substantial entomological inoculation rate (EIR) of 339 infective bites per person per year, peaking during the rainy season. This rate, primarily due to *An. gambiae*, was influenced by a heavy rainy season, which led to a resurgence at the start of the rainy season [20]. Furthermore, studies by Adja *et al.* [1] and Zoh *et al.* [36] aimed at updating vector transmission data in Bouaké after the 2002–2011 military-political crisis in Côte d’Ivoire. They showed that *An. gambiae* remained the primary malaria vector in Bouaké, exhibiting high activity during the rainy season.

Efforts to control and prevent malaria included the mass distribution of long-lasting insecticide-treated bed nets (LLINs), which were used as a protective measure. Led by the Ministry of Health and Public Hygiene through the National Malaria Control Program (NMCP), these efforts aimed to cover the entire population. On the other hand, from 2002 to 2011, the crisis triggered a significant exodus of residents from the city. Several neighborhoods were abandoned, which left the natural environment unmaintained and halted all vector control activities. The return of residents to these neighborhoods required the implementation of research programs to collect new data on malaria transmission parameters and distribution. This study is significant because it targets the period most conducive to the development of malaria vectors in Bouaké. Its objective is to assess the level of vector-borne malaria transmission by determining entomological parameters in Bouaké’s three health district neighborhoods.

## Materials and methods

### Study area

Bouaké (7°40’476’’N, 5°1’38’’W) is a city in central Côte d’Ivoire located 372 km from Abidjan. Spanning 312 km² and home to over 500,000 people, Bouaké is the second-largest city in Côte d’Ivoire after Abidjan, the economic capital. It is situated in an ecological transition zone (forest/ savanna). Rainfall generally occurs over an eight-month period, from March to October [16]. The annual average rainfall ranges from 1,000 to 1,200 mm. Temperatures vary little throughout the year, averaging from 28 to 32°C. Annual relative humidity ranges from 75% to 90%. Due to its hilly terrain, the city has several kilometers of marshy strips. The urban area is crisscrossed by numerous small waterways spaced 500 to 800 meters apart. Consequently, all neighborhoods in the city, except for the commercial center, are crossed by or bordered by strips of wet lowlands. Bouaké is divided into three health districts: Northwest, Northeast, and South. The study area, study design, and local malaria epidemiology have already been described in detail [33]. For each district, three neighborhoods were selected. In the northwest district, the selected neighborhoods are N’Gattakro, Djézoukouamékro, and Dar-Es-Salam. In the northeast district, the selected neighborhoods are Sokoura, Belle Ville, and Attienkro. In the south district, the selected neighborhoods are Kennedy, Air France, and N’Gouatanoukro (Fig. 1). In this region, malaria transmission is intense. *Plasmodium falciparum* is the main parasite species [32], and *An. gambiae* s.l. is the main vector [24].

**Figure 1:** Bouaké area showing the study sites (source: CNTIG, 2018 / Palevalute, 2018 – Produced by: AZONGNIBO Mardoché, 2024)

### Field mosquito sampling

Sampling was conducted in urban areas during the rainy season, from June 10 to 29, 2016, in the three health districts of Bouaké. The selection of capture sites began with a preliminary visit to these districts. Using a city map and taking into account the environmental characteristics of the districts, three neighborhoods per district were selected, then each neighborhood was divided into different lots called capture sites. Mosquito collection was conducted using the human-based trapping method (HBT). Over three consecutive nights (from 5:00 PM to 9:00 AM), the mosquito collection was conducted at four capture sites per neighborhood, with two collectors, one inside and one outside the selected dwellings (totaling 216 capture sessions).

These captures were carried out by two teams of volunteers of both genders (local residents). The first team worked from 5:00 PM to midnight, and the second from midnight to 9:00 AM. Captured mosquitoes were grouped hourly by site and stored in separate bags. Using this strategy, the aggressive density or density of anthropophilic *Anopheles* per person per unit of time (night) was estimated and used to calculate EIRs. All collected mosquitoes were morphologically identified using Edwards’ identification key for Culicidae [15] and that for female Afrotropical *Anopheles* [9]. The ovaries of female *Anopheles* were dissected, and the degree of coiling of the ovarian tracheoles was observed to determine their parity status based on Detinova’s criteria [11]. All collected female *Anopheles* were stored individually in Eppendorf tubes containing a desiccant, labeled with the study site name, collection point, and date. They were stored at -20 °C for subsequent analysis at the Pierre Richet Institute laboratory in Bouaké.

### Laboratory processing of mosquitoes

An enzyme-linked immunosorbent assay (ELISA) was used to test for the presence of *P. falciparum* circumsporozoite protein (CSP). This parasite is the predominant species, accounting for more than 95% of malaria cases. The heads and thoraxes of female *Anopheles* were used for the test. The head and thorax were separated from the rest of the body and homogenized in a blocking buffer containing casein at 0.5%, NaOH 0.1 N, and 1× PBS. The procedures were based on those of Burkot *et al.* [6] and Wirtz *et al.* [35]. A sample was considered positive if the optical density (OD) value was greater than twice the average OD of the four negative control wells (uninfected mosquitoes) on the ELISA plate.

### Entomological parameters and statistical analysis

Quantitative data collected during entomological surveys were entered into Excel. The dataset was then transferred to STATA version 8.0 for analysis. Aggressive density, infestation rate, and parturition rate were calculated using standard methods previously described by Fontenille *et al.* [19]. The Kruskal-Wallis test was used to compare mean aggressive densities. The chi² test was used to compare infestation and parturition rates. All analyses were performed with a 5% confidence interval.

## Results

### Culicidae fauna

A total of 29,528 mosquitoes were captured in the three districts by 216 men-nights, representing a nuisance of 136.7 bites/person/night (b/p/n). These mosquitoes belong to four genera (*Anopheles*, *Culex*, *Mansonia*, and *Aedes*), all of which were found in each district.

The captures yielded 6,104 mosquitoes in the Northwest district, comprising 8 species (*An. gambiae*, *An. pharoensis*, *Cx. quinquefasciatus*, *Cx. nebulosus*, *Cx. annulioris*, *Cx. cinereus*, *Man. africana* and *Ae. aegypti*), with a culicide nuisance index of 84.7 b/p/n. In the district’s three neighborhoods, the Culicidae nuisance indices were 70.7, 85.9, and 97.6 b/p/n, respectively in Dar-Es-Salam, Djezoukouamekro, and N’Gattakro. The genus *Culex* was predominant, accounting for 95.2% of the fauna, followed by the genus *Anopheles* with 4.3% (Table I). *Cx. quinquefaciatus* was the predominant species, accounting for 97.7%.

**Table I:** Culicidae fauna of the sampled sites in the Bouaké health districts

**Genera**	**Species**	**Nortdwest Bouaké**			**Nortdeast Bouaké**			**South Bouaké**		
		Dar-Es-Salam n (%)	Djézoukouamékro n (%)	N'Gattakro n (%)	Sokoura n (%)	Attienkro n (%)	Belle Ville n (%)	Air France n (%)	Kennedy n (%)	N'Goutanoukro n (%)
Anopheles	*An. gambiae*	35 (2,1)	185 (9)	41 (1,7)	76 (1,7)	585 (15,8)	456 (24,8)	21 (0,4)	987 (32,4)	28 (0,5)
	*An. pharoensis*	-	3 (0,1)	-	1 (0,02)	6 (0,2)	-	-	8 (0,3)	-
	*An. ziemanni*	-	-	-	-	-	-	-	1 (0,0)	-
**Total 1 Anopheles**		**35 (2,1)**	**186 (9,1)**	**41 (1,7)**	**77 (1,7)**	**588 (16)**	**464 (24,8)**	**21 (0,4)**	**996 (32,7)**	**28 (0,5)**
Culex	*Cx. quinquefasciatus*	1 644 (96,9)	1 868 (90,5)	2 271 (96,9)	4 464 (98,2)	1 592 (43,0)	1 320 (71,7)	4 714 (99,4)	1 564 (51,3)	5 512 (99,5)
	*Cx. nebulosus*	6 (0,4)	1 (0,05)	-	-	-	7 (0,4)	-	18 (0,6)	-
	*Cx. annulioris*	-	1 (0,05)	2 (0,1)	-	1 (0,03)	-	-	1 (0,03)	-
	*Cx. decens*	-	-	-	-	8 (0,2)	5 (0,3)	-	-	-
	*Cx. cinereus*	8 (0,5)	3 (0,1)	6 (0,3)	3 (0,1)	-	7 (0,4)	-	-	1 (0,02)
	*Cx. ingrami*	-	-	-	-	-	25 (1,4)	-	-	-
	*Cx. tigripes*	-	-	-	-	-	6 (0,3)	-	-	-
**Total 2 Culex**		**1 658 (97,7)**	**1 873 (90,8)**	**2 276 (97,2)**	**4 467 (98,3)**	**3 701 (43,2)**	**1 370 (74,4)**	**4 714 (99,4)**	**1 583 (51,9)**	**5 513 (99,5)**
Mansonia	*Man. africana*	-	2 (0,1)	-	1 (0,02)	333 (9,1)	1 (0,1)	-	94 (3,1)	-
	*Man. uniformis*	-	-	-	-	840 (22,7)	12 (0,7)	-	55 (1,8)	-
**Total 3 Mansonia**		-	**2 (0,1)**	-	**1 (0,0)**	**1 173 (31,7)**	**13 (0,7)**	-	**149 (4,9)**	-
Aedes	*Ae. aegypti*	4 (0,2)	-	24 (1,0)	-	338 (9,1)	2 (0,1)	7 (0,1)	319 (10,5)	1 (0,02)
	*Ae. vittatus*	-	-	-	-	-	-	-	2 (0,1)	-
	*Ae. palpalis*	-	-	-	-	-	-	-	1 (0,03)	-
	*Ae. africanus*	-	-	-	-	1 (0,03)	-	-	-	-
**Total 4 Aedes**		**0,2**	-	**24 (1,0)**	-	**339 (9,2)**	**2 (0,1)**	**7 (0,1)**	**322 (10,6)**	**1 (0,02)**
**TOTAL**		**1 697**	**2 063**	**2 344**	**4 545**	**3 704**	**1 841**	**4 742**	**3 003**	**5 542**

A total of 10,090 mosquitoes was collected in the Northeast district. The genus *Culex* was predominant (73.7%), followed by the genera *Mansonia* (11.8%) and *Anopheles* (11.1%). Captures on humans identified 13 species, with *Cx. quinquefasciatus* being the most common (73.1% of the captures). The average Culicidae nuisance index was 140.1 b/p/n, including 189.4 b/p/n in Sokoura, 154.3 b/p/n in Attienkro, and 76.7 b/p/n in Belle Ville. In all three neighborhoods, the genus *Culex* was predominant, accounting for 98.3%, 74.4%, and 43.2% in Sokoura, Belle Ville, and Attienkro, respectively. It was followed by the genus *Mansonia* (31.7% in Attienkro) and the genus *Anopheles*, with proportions of 24.8% and 1.7% in Belle Ville and Sokoura, respectively (Table I).

In the South district, 13,334 mosquitoes were captured. As in the other two districts, the *Culex* genus was predominant, accounting for 88.6%. Among the 12 collected species, *Cx. quinquefasciatus* was predominant, accounting for 88.4% of the mosquitoes. The district’s average nuisance index was 185.2 b/p/n. Nuisance indices were 197.6, 230.9, and 127.1 b/p/n in Air France, N’Gouatanoukro, and Kennedy, respectively (Table I). *Culex* was the most abundant genus in each of the district’s three neighborhoods, accounting for 99.5%, 99.4%, and 51.9% of the total in N’Gouatanoukro, Air France, and Kennedy, respectively. In this district, the genus *Anopheles* accounted for between 0.4% and 32.7% of the Culicidae.

### Biting behavior of *An. gambiae* s.l.

The simultaneous collection of mosquitoes inside and outside dwellings made it possible to determine the feeding habits of female *An. gambiae* s.l. (Table II). The endophagous or exophagous rate is the proportion of female, malaria-vectoring mosquitoes that bite inside or outside dwellings. It is expressed as a percentage. Of the 2,414 female *An. gambiae* s.l. captured in the city of Bouaké, 1,269 (52.6%) were captured indoors and 1,145 (47.4%) outdoors. In the Northwest health district, *An. gambiae* females feed both indoors and outdoors. Exophagous feeding was observed in Dar-Es-Salam and Djézoukouamékro. Although endophagous feeding is generally observed in the Sokoura and Air France neighborhoods, *An. gambiae* females feed inside and outside dwellings.

**Table II:** Biting behavior of female *An. gambiae* s.l. in the three health districts of Bouaké

Districts	Neighborhoods	females captured (%)		Predominant status
		In n (%)	Out n (%)	
**Northwest Bouaké**	N'Guattakro	31 (75,60)	10 (24,4)	Endophagy
	Dar-Es-Salam	12 (34,3)	23 (65,7)	Exophagy
	Djézoukouamékro	84 (45,9)	99 (54,1)	Exophagy
**TOTAL**		**127 (49)**	**132 (51)**	**Endophagy**
**Northeast Bouaké**	Sokoura	38 (50)	38 (50)	Endo-exophagy
	Belle Ville	261 (56,2)	203 (43,8)	Endophagy
	Attienkro	306 (52,6)	276 (47,4)	Endophagy
**TOTAL**		**605 (53,9)**	**517 (46,1)**	**Endophagy**
**South Bouaké**	Air France	10 (50)	10 (50)	Endo-exophagy
	Kennedy	497 (52,9)	443 (47,1)	Endophagy
	N'Goutanoukro	11 (42,3)	15 (57,7)	Exophagy
**TOTAL**		**518 (52,5)**	**468 (47,5)**	**Endophagy**

n: Headcount; In: Inside; Out: Outside; (%): Percentage of females in the total catch

### Aggressive density of *An. gambiae* s.l.

*An. gambiae* s.l. was the only identified malaria vector in the three districts of Bouaké. Its average density was 11.2 b/p/n across the entire city (Table III).

**Table III:** Aggressive density of *An. gambiae* s.l. in the three health districts of Bouaké

Districts	Neighborhoods	ma (b/h/n)	CI
**Northwest Bouaké**	N'Gattakro	1,7 (41)	0,8 - 2,6
	Dar-Es-Salam	1,5 (35)	0,5 - 2,4
	Djézoukouamékro	7,7 (185)	4,1 - 11,3
**Average**	**3,6 (261)**	**2,2 - 5**	
**Northeast Bouaké**	Belle Ville	19 (456)	13,7 - 24,3
	Attienkro	24,4 (585)	16,6 - 32,2
	Sokoura	32 (76)	1,8 - 4,5
**Average**	**15,5 (1 117)**	**11,8 - 19,2**	
**South Bouaké**	Air France	0,9 (21)	0,5 - 1,3
	Kennedy	41,1 (987)	28,5 - 53,8
	N'Goutanoukro	1,2 (28)	0,5 - 1,8
**Average**	**14,4 (1 036)**	**8,4 - 20,4**	

ma. aggressivity in bites/person/night (b/p/n); CI: confidence interval (95%)

In the Northwest district, the density was 3.6 b/p/n. Among the neighborhoods in this district, the biting density in Djézoukouamekro (7.7 b/p/n) was significantly higher than in N’Gattakro (1.7 b/p/n) and Dar-Es-Salam (1.5 b/p/n) (p = 0.002).

The biting density of *An. gambiae* s.l. in Northeast Bouaké was 15.5 b/p/n. The biting density of the vector in Sokoura (3.2 b/p/n) was significantly lower than in Attienkro (24.4 b/p/n) and Belle Ville (19 b/p/n) (p < 0.10^-^⁴).

In the South district, *An. gambiae* aggressivity was 14.4 b/p/n. The biting density in Kennedy (41.1 b/p/n) was significantly higher (p < 0.0001) than in Air France (0.9 b/p/n) and N’Gouatanoukro (1.2 b/p/n). *An. gambiae* s.l. aggressivity in Northwest Bouaké was significantly lower (p < 0.0001) than in the other two districts, where densities did not vary significantly.

### *Parturition rate of* An. gambiae *s.l.*

A total of 809 *An. gambiae* females (216 in the Northwest District, 377 in the Northeast District, and 216 in the South District) were dissected on site, yielding an estimated parturition rate of 93.8%. The rate was estimated to be 92.6% and 95% inside and outside dwellings, respectively (Table IV)

**Table IV:** Parturition rate of female *An. gambiae* s.l. collected in the three health districts of Bouaké

Districts	Neighborhoods	Number of tested		Number of parous females		Parturition rate (%)		Total PR (%)
		In n (%)	Out n (%)	In n (%)	Out n (%)	In n (%)	Out n (%)		
**Northwest Bouaké**	Dar-Es-Salam	11	19	11	19	100	100	100
	Dézoukouamékro	83	80	77	73	92,8	91,3	92
	N'Gattakro	15	8	15	8	100	100	100
**TOTAL**		109	107	103	100	94,5	93,5	94
**Northeast Bouaké**	Sokoura	30	40	28	34	93,3	85	88,6
	Attienkro	68	86	68	86	100	100	100
	Belle Ville	97	56	83	55	85,6	98,2	90,2
**TOTAL**		195	182	179	175	91,8	96,2	94,9
**South Bouaké**	N'Goutanoukro	13	14	8	11	61,5	78,6	70,4
	Air France	9	13	7	9	77,8	69,2	72,7
	Kennedy	65	102	65	102	100	100	100
**TOTAL**		87	129	80	122	92	94,6	93,5

In the Northwest health district, the parturition rate was 94% of 216 female *An. gambiae*. This rate was nearly identical inside and outside of dwellings, with respective proportions of 94.5% and 93.5%. In the Dar-Es-Salam and N’Gattakro, the parturition rates were 100% and 92% in Djézoukouamékro.

A total of 377 *An. gambiae* females were dissected in the Northeast district, with an estimated parturition rate of 94.9%. In this district, the parturition rates inside and outside dwellings were 91.8% and 96.2%, respectively. In Attienkro, the parturition rate was 100%, while in Sokoura and Belle Ville, it was 88.6% and 90.2%, respectively. In the South district, 216 *An. gambiae* females were dissected, yielding an estimated parturition rate of 93.5%. The parturition rates of *An. gambiae* females dissected inside and outside dwellings were 92% and 94.6%, respectively. In Kennedy, the rate was 100%. N’Gouatanoukro and Air France had nearly identical rates, estimated at 70.4% and 72.7%, respectively.

### Infection and entomological inoculation rates

Of the 807 female *Anopheles* mosquitoes tested throughout Bouaké, 24 were infected. The average infection rate was 3%, resulting in an EIR of 0.33 b/p/n (Table V). The infestation rate in the Northwest District was 4.2%. There was no significant difference between this rate and the rates in the Northeast (2.9%; p = 0.40) and South (1.8%; p = 0.14) districts.

**Table V:** Infection rate and entomological inoculation rates

Districts	Neighborhoods	Number tested (Number infected)			EIR (ib/p/n)
		In n (%)	Out n (%)	Total	
**Northwest Bouaké**	N'Gattakro	14 (1)	8 (1)	22 (2)	0,15
	Djezoukouamékro	85 (5)	78 (1)	163 (6)	0,28
	Dar-Es-Salam	11 (0)	19 (1)	30 (1)	0,05
**TOTAL**		**110 (6)**	**105 (3)**	**215 (9)**	**0,15**
**Northeast Bouaké**	Attienkro	85 (3)	63 (1)	148 (4)	0,67
	Sokoura	38 (1)	35 (0)	73 (1)	0,04
	Belle Ville	106 (4)	52 (2)	158 (6)	0,72
**TOTAL**		**229 (8)**	**150 (3)**	**379 (11)**	**0,45**
**South Bouaké**	Kennedy	70 (1)	99 (2)	169 (3)	0,45
	Air France	12 (0)	11 (1)	23 (1)	0,004
	N'Gouattanoukro	10 (0)	17 (0)	27 (0)	0
**TOTAL**		**92 (1)**	**127 (3)**	**219 (4)**	**0,22**
**TOTAL BOUAKÉ**		**431 (15)**	**382 (9)**	**813 (24)**	**0,33**

Number tested: number of females tested; Number infected: number of females infected; EIR: entomological inoculation rate;

In the Northwest District, nine female *Anopheles* mosquitoes were found to be infected with *P. falciparum* out of 215 tested, representing an infestation rate of 4.2%. Infestation rates were 3.7% in Djézoukouamékro, 3.33% in Dar-Es-Salam, and 9.1% in N’Gattakro.

These rates did not differ significantly (p = 0.41). Analyses of *Anopheles* captured inside and outside of dwellings revealed no significant differences in infestation rates among the neighborhoods of Djézoukouamékro, N’Gattakro, and Dar-Es-Salam (p = 0.11, p = 0.67, and p = 0.43, respectively). The EIR in this district is 0.15 b/p/n. However, the rate in the Djézoukouamékro neighborhood was the highest (0.28 b/p/n). The rates for N’Gattakro and Dar-Es-Salam were 0.15 b/p/n and 0.05 b/p/n, respectively.

Of the 379 females tested, 11 were infected in the Northeast district, representing an infestation rate of 2.9%. The infestation rates in Belle Ville, Attienkro, and Sokoura were 3.8%, 2.7%, and 1.3%, respectively. These rates did not differ significantly (p = 0.59). There was no significant difference in infestation rates obtained inside and outside homes in Belle Ville (p = 0.98), Sokoura (p = 0.33), and Attienkro (p = 0.47). The EIR was low in Sokoura (0.04 b/p/n), compared to Attienkro and Belle Ville, where the EIRs were 0.67 b/p/n and 0.72 b/p/n, respectively.

Testing of the 219 female *Anopheles* mosquitoes collected in the South District confirmed infection in four mosquitoes, representing an infestation rate of 1.8%. The average infestation rate of females captured in Kennedy was 1.8%, which is comparable to the rate estimated in Air France: 4.3% (p = 0.41). Females captured in N’Gouatanoukro showed no infection. In Kennedy and Air France, the infestation rates of females collected inside and outside dwellings were similar (p = 0.28). The Kennedy neighborhood recorded a high EIR of 0.45 b/p/n.

## Discussion

The inventory of anthropophilic Culicidae fauna reveals a high diversity and abundance of mosquitoes during the rainy season. Using the human-trap method, four genera and 16 species of mosquitoes were collected across all three districts of Bouaké. The variety of habitats in this area, which offer specific breeding sites conducive to the development of each species, can explain this diversity of Culicidae in all districts: rice paddies, puddles, ponds, swamps, standing water in old containers, etc. [1]. Culicidae population size is closely linked to the functioning and dynamics of larval habitats. Breeding sites [5] (e.g., ponds, tires, and containers) change over time (e.g., seasons and rainfall) and space (e.g., urban versus rural areas) in terms of number, type, and larval productivity. Additionally, the influence of physical factors (e.g., temperature, oxygen, and pH) on the reproduction of disease-vector mosquitoes has to be considered [25].

HBT’s entomological results showed that the anthropophilic Culicidae fauna in Bouaké was dominated by *Cx. quinquefasciatus,* as Adja *et al.* observed [1]. The predominance of this species is often associated with urban environments [3]. In Bouaké, it is a significant nuisance to the population. Studies conducted by Klinkenberg *et al.* in Accra [23] and by Fillinger *et al*. in Dar es Salaam [18] are consistent with these observations. In the three health districts, the *Anopheles* fauna was predominantly dominated by *An. gambiae* s.l., the primary malaria vector. This species ranked second among the Culicidae in terms of capture frequency across all districts, behind *Cx. quinque-fasciatus*. This finding is consistent with the work of Assouho *et al.* [3], which confirms the species’ presence in urban areas. The notable prevalence of *An. gambiae* is associated with human activities that create permanent or semi-permanent breeding sites, such as crop irrigation, rice paddies, and urbanization [7,25,27,31]. Furthermore, the presence of *Anopheles* is associated with environmental degradation caused by war. They are often found in areas with degraded environmental conditions, which wars can exacerbate by creating larval breeding sites (e.g., stagnant water due to destruction or lack of water management), destroying sanitation infrastructure, and disrupting control programs. This promotes the proliferation of malaria-carrying mosquitoes. This vicious cycle exacerbates health problems in conflict zones [2].

Overall, investigations reveal that mosquitoes tend to feed more inside homes than outside in both the northern and southern districts. This endophagic behavior persists despite the introduction of LLINs into homes. This behavior could be explained by high resistance to the pyrethroid insecticides used to treat LLINs. However, it also raises questions about how these nets are actually used by the population to avoid mosquito bites and prevent malaria. Furthermore, this vector behavior appears well-adapted to the nocturnal habits of humans, which are influenced by the rainy season. This adaptation of mosquito biting behavior to human nocturnal habits in urban areas is similar to observations made by Salako *et al.* in Benin [30]. In contrast, malaria vector populations in the Northwest District tend to feed both indoors and outdoors, reflecting a potential change in vector behavior.

The density of *An. gambiae* s.l. was generally higher in the Northeast and South districts than in the Northwest district. This difference may be due to the development of low-lying areas for irrigated rice cultivation (Attienkro and Belle Ville) and vegetable farming (Kennedy), as well as environmental degradation caused by the war. In Kennedy, the average aggressive density of 41.2 b/p/n for *An. gambiae* was significantly higher than the 18.5 b/p/n reported by Dossou-Yovo *et al.* during the same period [14]. Furthermore, other studies have revealed higher mosquito densities in irrigated agricultural areas (e.g., rice paddies and vegetable gardens) than in neighboring uncultivated areas [12,14,22]. Due to their geographic location, the Djézoukouamékro, N’Gattakro, and Dar-Es-Salam sites in the Northwest district largely represent the center of the city, which explains the low biting density in this district. These observations confirm previous studies showing a reduction in *An. gambiae* density from the outskirts to the city center [4,13]. Mosquito populations in the *An. gambiae* s.l. complex, which carry malaria, are more abundant in peripheral areas (rural or peri-urban). These populations decrease progressively as one approaches the city center [2], likely due to environmental differences (fewer breeding sites, increased urbanization, improved water management, etc.) or malaria control strategies.

The parturition rates observed during this study period were very high in the three districts. This indicates significant aging of the *Anopheles* population and a high proportion of potentially dangerous female mosquitoes, as they are most often infectious and have more time to come into contact with humans before dying. The proportion of parous females is 100% in the Kennedy, Dar-Es-Salam, and Attienkro neighborhoods. Therefore, the proportion of the *An. gambiae* capable of transmitting malaria is identical for these neighborhoods spread across the city’s three districts. Furthermore, Dossou-Yovo *et al.* [14] reported an average parturition rate of 71.7% for *An. gambiae* in Kennedy during the rainy season, whereas our study shows a higher rate of 100% for the same species and the same rainy season approximately 25 years later.

The infestation rate is very high across all districts and is likely due to high parity rates. Indeed, the older the mosquitoes are, the greater their likelihood of harboring the parasite. This could result in continuous and intense malaria transmission. The endemicity of malaria in Bouaké, with a resurgence during the rainy season, is similar to that typically described in Côte d’Ivoire [3]. The EIR of 0.33 b/p/n (or 9.9 b/p/month) observed in this study was lower than the 1.7 b/p/n (or 51 b/p/month) reported by Dossou-Yovo *et al.* [14]. The situation in Bouaké is highly heterogeneous, as is the case in most African cities [8,14,34]. Each neighborhood is unique with its own influences, which may depend on human factors or the natural characteristics of the environment. The diversity of situations observed clearly illustrates the importance of local, and sometimes micro-ecological, conditions on the intensity of malaria transmission. The risk to be infected by *Plasmodium* remains high in Bouaké.

## Conclusion

This study, conducted in the city of Bouaké, revealed heterogeneous malaria transmission caused by *An. gambiae*. Its endophagous behavior makes *An. gambiae* the sole malaria vector in the city’s various neighborhoods. This vector predominates in the Northeast District, specifically in the Attienkro and Belle Ville neighborhoods. The Kennedy neighborhood has the highest *An. gambiae* population and is the source of the vector’s presence in the South District.

Of all the districts sampled in Bouaké, the Northeast District had the highest risk of entomological transmission, particularly in the Attienkro and Belle Ville neighborhoods. This malaria transmission risk level is twice that of the South District and three times that of the West District. Therefore, it warrants special attention within the framework of malaria control programs in this city. Furthermore, control measures, such as the use of larvicides, should be encouraged among individuals engaged in activities conducive to the proliferation of malaria vectors, such as vegetable farming or rice cultivation.

## Conflict of interest, funding, and ethical principles

This research was funded by the IRD through the JEAI EVAPALCI project. The authors declare no conflict of interest. The study received approval from the National Ethics and Research Committee of Côte d’Ivoire (June 2014; No. 41/MSLS/CNER-dkn) as well as from the health authorities of the city of Bouaké. Permission to conduct the survey was obtained from neighborhood leaders and homeowners whose properties served as collection sites. Additionally, community consent was obtained in advance at all sites. Volunteer mosquito collectors gave their verbal consent before participating in the study. They also underwent regular medical checkups and received preventive treatment against malaria in accordance with the recommendations of the NMCP of Côte d’Ivoire. All were vaccinated against yellow fever.

## Acknowledgments

We express our gratitude to the Bouaké Regional Health Directorate for the opportunity it provided by authorizing us to conduct our survey in the city’s various neighborhoods. We are very grateful to the leaders of the sampled neighborhoods as well as to all the mosquito collectors. Finally, we also thank Mr. Jean-Luc Amany for his support during the completion of this work, and the IRD through the JEAI EVAPALCI project for financial support.
